# Genetic analysis of hybridization and introgression between wild mongoose and brown lemurs

**DOI:** 10.1186/1471-2148-9-32

**Published:** 2009-02-05

**Authors:** Jennifer Pastorini, Alphonse Zaramody, Deborah J Curtis, Caroline M Nievergelt, Nicholas I Mundy

**Affiliations:** 1 Anthropologisches Institut, Universität Zürich, Winterthurerstrasse 190, 8057 Zürich, Switzerland; 2 Centre for Conservation and Research, 35 Gunasekara Gardens, Nawala Road, Rajagiriya, Sri Lanka; 3 Département des Sciences de la Terre, Université de Mahajanga, Faculté des Sciences, Dépt. de Biologie Animale, B.P. 652, Mahajanga 401, Madagascar; 4 Department of Anthropology & Geography, School of Social Sciences & Law, Oxford Brookes University, Gipsy Lane, Headington, Oxford, OX3 0BP, UK; 5 Department of Psychiatry, University of California at San Diego, 9500 Gilman Drive, La Jolla, CA 92093-0603, USA; 6 Department of Zoology, University of Cambridge, Downing Street, Cambridge, CB2 3EJ, UK

## Abstract

**Background:**

Hybrid zones generally represent areas of secondary contact after speciation. The nature of the interaction between genes of individuals in a hybrid zone is of interest in the study of evolutionary processes. In this study, data from nuclear microsatellites and mitochondrial DNA sequences were used to genetically characterize hybridization between wild mongoose lemurs (*Eulemur mongoz*) and brown lemurs (*E. fulvus*) at Anjamena in west Madagascar.

**Results:**

Two segments of mtDNA have been sequenced and 12 microsatellite loci screened in 162 brown lemurs and mongoose lemurs. Among the mongoose lemur population at Anjamena, we identified two F1 hybrids (one also having the mtDNA haplotype of *E. fulvus*) and six other individuals with putative introgressed alleles in their genotype. Principal component analysis groups both hybrids as intermediate between *E. mongoz *and *E. fulvus *and admixture analyses revealed an admixed genotype for both animals. Paternity testing proved one F1 hybrid to be fertile. Of the eight brown lemurs genotyped, all have either putative introgressed microsatellite alleles and/or the mtDNA haplotype of *E. mongoz*.

**Conclusion:**

Introgression is bidirectional for the two species, with an indication that it is more frequent in brown lemurs than in mongoose lemurs. We conclude that this hybridization occurs because mongoose lemurs have expanded their range relatively recently. Introgressive hybridization may play an important role in the unique lemur radiation, as has already been shown in other rapidly evolving animals.

## Background

Hybridization among animals has traditionally been viewed as an unusual event. However, recent genetic studies have shown that it occurs more commonly than originally believed [1,2]. Hybridization may occur due to human impact, such as between domestic or captive species, wild and domestic species [3,4] or between introduced and native species [5,6]. Natural hybridization has been found across the animal kingdom, including insects [7], fish [8], amphibians [9], birds [10,11], carnivores [12], and monkeys [13-15]. Among the Malagasy lemurs, a few cases of hybridization between subspecies have been reported in the wild [16-19].

Harrison [20] defined hybridization as "the interbreeding of individuals from two populations, or groups of populations, which are distinguishable on the basis of one or more heritable characters." Hybridization occurs when there are incomplete reproductive barriers between two taxa. There are many possible evolutionary outcomes of hybridization: 1) The two hybridizing taxa may merge, 2) reproductive barriers may be reinforced between the parental taxa, 3) transfer of genetic material into one or both parental taxa may occur, which might facilitate adaptive evolution 4) a new species of hybrid origin may be formed, or 5) the hybrid zone may become established without any major impact on the parental taxa [21,22]. Therefore, studies of natural hybrids and their genetic composition can shed new light on issues concerning reproductive barriers, survivorship and fitness of hybrids and give important insights into evolutionary processes and the adaptation of species.

The Malagasy lemurs are a spectacular example of adaptive radiation and provide an ideal model for studies of evolutionary diversification [23]. Currently, 71 lemur species and subspecies are recognized, which are distributed across 15 genera in 5 families [24]. Madagascar's isolation, diverse climate, geology and vegetation provide conditions equating to a natural experiment in taxonomic diversification. Hybridization has been found to play a major role in rapid radiations such as Darwin's finches [11], East African cichlid fishes [8], Hawaiian crickets [25] and passion-vine butterflies [7]. However, little is known about interspecific gene flow or its role in the radiation of lemurs.

During a field study on mongoose lemurs (*Eulemur mongoz*) at Anjamena in western Madagascar an animal was observed which exhibited a pelage coloration intermediate between that of red-fronted brown lemurs (*E. fulvus rufus*) and mongoose lemurs [26]. This phenotypic variation was the first sign that interspecific hybridization might be occurring at this site. When sequencing some *E. f. rufus *individuals from the same locality for a phylogenetic study of brown lemurs [27], one individual was found to have the mtDNA haplotype of *E. mongoz *[28]. This hardened the suspicion of Anjamena being a hybrid zone between two taxonomically well accepted *Eulemur *species.

**Figure 1:**
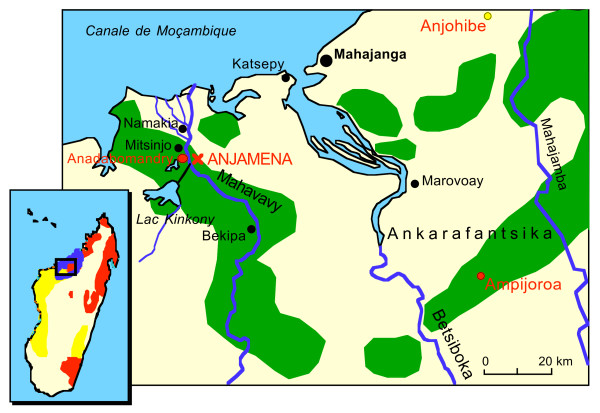
**Location of Anjamena (red cross) where *E. mongoz *and *E. fulvus *hybridize**. Two additional sample locations (Anadabomandry and Ampijoroa) and one fossil site (Anjohibe) are labelled in red. Green areas are forests. The inset shows the distribution of *E. mongoz *(blue border), *E. f. rufus *(yellow) and other *E. fulvus *subspecies (red).

Mongoose lemurs and brown lemurs are two of five species in the genus *Eulemur*, in the family Lemuridae. *E. mongoz *occurs in the west of Madagascar. Brown lemurs are divided into 6 subspecies, which together range over a large area of Madagascar, excluding the South of the island. Notably, *E. fulvus *and *E. mongoz *are the only two species of lemurs found outside Madagascar, and are thought to have been introduced to the Comoros by humans. In Madagascar, East of the Betsiboka river (Figure 1), mongoose lemur distribution overlaps with that of *E. f. fulvus*. To the west of the Betsiboka river *E. mongoz *is sympatric with *E. f. rufus*. The ranges of both brown lemur subspecies are much larger than the relatively small area mongoose lemurs occupy. *E. mongoz *is identified as 'vulnerable' in the IUCN red list of threatened species, while *E. fulvus *is classified as at 'lower risk' of extinction [29]. Habitat destruction and forest fragmentation are the main threats to both taxa.

Mongoose lemurs at Anjamena live in small family groups of 2–6 individuals, consisting of an adult pair and associated offspring. Group composition remains relatively constant, with changes being limited to births and emigration of subadult individuals [26]. Mongoose lemurs are cathemeral throughout the year with shifts towards more diurnal activity in the wet season and more nocturnal activity in the dry season [30]. *E. mongoz *is predominantly frugivorous, its diet being supplemented by leaves, flowers, and nectar [31].

The most important potential competitor for mongoose lemurs at Anjamena is *E. f. rufus*, as it not only shares food resources (fruit and leaves) with *E. mongoz *but also exhibits a similar activity pattern [26]. Brown lemurs at Anjamena live in large groups of up to nine individuals with a variable sex and age composition [32]. In 1995, population density of *E. f. rufus *(121 individuals per km^2^) in the vicinity of Anjamena was much higher than that of *E. mongoz *(45 individuals per km^2^) [32]. Both *E. mongoz *and *E. fulvus *exhibit sexual dichromatism. The pelage coloration of brown lemur males and females is very different from that of mongoose lemur males and females (for illustrations see [24]). At Anjamena, the body weight of adult brown lemurs (ca. 1700 g) is higher than that of adult mongoose lemurs (ca. 1200 g, pers. observation). Association between the two species has not been observed at Anjamena [26].

We conducted a comprehensive genetic analysis, including both nuclear microsatellites and mitochondrial DNA (mtDNA) sequences of *E. mongoz *and *E. fulvus*, to gain insights into the genetic composition of the two species, and to assess hybridization between them in Anjamena. Microsatellites show a high degree of length polymorphism, which makes it possible to obtain pedigree-level characterization of populations as well as to detect introgression of alleles between species. Analysis of mtDNA sequences allows direct determination of matrilineal relatedness, which provides information on past hybrid events.

## Methods

### Study population

A total of 163 samples from mongoose lemurs (108) and brown lemurs (55) were collected from the wild and captivity (Table 1). The main study site Anjamena is situated in the riverine forests of the Mahavavy in western Madagascar (45°55'E, 16°03'S, Figure 1) from where a total of 38 samples of *E. mongoz *were collected. Five hair samples were collected in 1994 and 1995 during a behavioural study [30]. From August to September 1997, samples of 33 mongoose lemurs from 12 neighbouring groups and 8 brown lemurs from 7 groups were collected at Anjamena. In the same time period, 4 *E. mongoz *and 2 *E. fulvus *samples were collected from animals living near Anadabomandry across the Mahavavy river from Anjamena. Additionally, 2 mongoose lemur and 3 brown lemur samples from Ampijoroa (110 km east of Anjamena, separated by the river Betsiboka, Figure 1) were analysed. From the animals sampled in 1997, body weight was measured and fur coloration was recorded by taking photographs.

**Table 1:** Samples available from mongoose and brown lemurs

**Taxon**	**Location**	**# Samples**
*Eulemur mongoz*	Anjamena	38
	Anadabomandry	4
	Ampijoroa	2
	Madagascar (unknown)	1
	Captivity	63

*E. fulvus rufus*	Anjamena	8
	Anadabomandry	2
	Other	12

*E. f. fulvus*	Various zoos/locations	14
*E. f. albifrons*	Various zoos/locations	10
*E. f. sanfordi*	Tsimbazaza Zoo	3
*E. f. collaris*	Banham Zoo, pet in Fort Dauphin	2
*E. f. albocollaris*	Strasbourg, Tsimbazaza Zoo	4

A total of 63 blood, hair or tissue samples were collected from mongoose lemurs in captivity. The origin of the captive mongoose lemur population is not entirely known, but some founder animals originated from the Comoro islands. One *E. mongoz *sample was of unknown origin. An additional 42 *E. fulvus *samples were acquired from various zoos, covering all brown lemur subspecies (Table 1). In addition, samples of 3 *E. coronatus*, 3 *E. rubriventer*, 3 *E. macaco macaco*, 3 *E. m. flavifrons *and 2 *Lemur catta *(all zoo animals) were sequenced for mtDNA [for more details see Additional file 1].

### Molecular methods

DNA was extracted from hair, blood or tissue samples using a standard phenol-chloroform extraction [33]. Approximately 10–100 ng template DNA was amplified in 20 μl (microsatellites) or 50 μl (mtDNA) reactions using 0.06 M Tris, 0.015 M (NH_4_)_2_SO_4_, 1.5 mM MgCl_2_, 0.78 M DMSO, 0.025 mM for each dNTP, 1 mM for each primer, and 0.5 U *Thermophilus aquaticus *(*Taq*) polymerase. Samples were amplified for 25–35 cycles, with denaturing at 95°C for 30 s, primer annealing at 50–65°C for 60 s, and extension at 72°C for 60 s, followed by a final extension for 5 min at 72°C. The number of cycles and/or the annealing temperature was changed as necessary, to optimize the PCR conditions for each locus.

A total of 12 microsatellite loci were used for this study. Locus Efr09J was originally isolated from *E. f. rufus *[34]. We performed the PCR of that locus with 60°C annealing and 25 cycles. To amplify locus Lc8, which was developed for *L. catta *[35], 55°C annealing temperature and 35 cycles were used. The other 10 loci were amplified using the PCR conditions described in [36]. PCR products were run on an ABI PRISM 377XL or 3730 automated DNA sequencer. Allele sizes were determined with GeneScan software (Applied Biosystems). For all loci, samples of 30 offspring and their known pairs of parents as well as 15 offspring with one available parent from the captive mongoose lemur colony were genotyped, allowing testing for Mendelian inheritance.

The 3' end of the tRNA^Thr ^gene, the complete tRNA^Pro ^gene and the 5' end of the control region (D-loop), as well as part of the NADH-dehydrogenase subunit 4 (ND4) gene were amplified and sequenced, using the primers listed in Table 2. The PCR products were electrophoresed in 2% agarose gels to estimate template concentration. The sequencing reactions were carried out with the BigDye Terminator Cycle Sequencing Ready Reaction Kit (Applied Biosystems), using 1 μl of terminator mix and 2 μl of 5× buffer for a 9 μl reaction. The completed sequencing reactions were cleaned of excess dyes with ethanol precipitation before being run on an automated DNA sequencer. All templates were sequenced in their entirety for both strands. The sequencing data was aligned with Sequencher™ 4.2.2 (Gene Codes Corporation).

**Table 2:** Forward (F) and reverse (R) primers used to amplify (A) and sequence (S) the two mtDNA fragments

**Primer**	**Sequence 5'-3'**	Map position*	**F/R**	**A**	**S**
283	tacactggtcttgtaaacc	15908–15926	F	x	x
LemurDLF1	aagcctagtccatacgcatataagc	16181–16205	F	x	x
LemurDLR2	ggtagattaagctacgatc	16303–16321	R	x	x
LemurDLR4	atctcYtatgtccttcaagcat	219–240	R	x	x
282	aaggctaggaccaaacct	651–668	R	x	
ND4F	taggaggataYggRataatacg	11472–11493	F	x	x
ND4R	atagatattagggtattttctcg	12053–12075	R	x	x

* Relative to human mtDNA [GenBank: V00662]

### Statistical analyses

For microsatellite data, CERVUS 3.0.3 [37] was used to calculate observed (H_O_) and expected (H_E_) heterozygosities, frequency estimates for null alleles and to test for deviations from the Hardy-Weinberg equilibrium. Additionally, non-exclusion probabilities for first and second parents on the 12 autosomal microsatellite loci and the probability for two random individuals having the same genotype were determined with CERVUS. GENEPOP 4.0.6 [38] was used to test for genotypic linkage disequilibrium in the wild mongoose lemurs at Anjamena. Bonferroni correction was applied to alpha to adjust for multiple testing of 12 loci. The population genetic structure was inferred by calculating F_is_, F_st _and R_st _estimates in GENEPOP and by performing an analysis of molecular variance (AMOVA) in Arlequin 3.11 [39]. Principal component analysis of the multilocus genotypes was carried out with GENETIX 4.05 [40]. STRUCTURE 2.2.2 [41] was used to infer the genetic structure of the populations based on microsatellite loci. The number K of populations was estimated using a burn-in period of 10,000 and 100,000 MCMC replicates, applying the admixture model and independent or correlated allele frequencies. The estimated membership coefficients Q for each individual in each cluster was calculated by STRUCTURE, in order to assign the individual to one or, if admixed, to several clusters.

The aligned mtDNA sequences were analysed with PAUP 4.0b10 [42] using maximum parsimony (2500 random addition heuristic search) and neighbor-joining (Kimura 2-parameter distances) methods. Gaps were considered as a fifth character state in parsimony analyses, whereas in neighbor-joining analyses they were treated as missing data. Bootstrap analyses of 1000 replicates (10 random addition heuristic searches each) for maximum parsimony and 2500 replicates for neighbor-joining were performed to examine the relative support of each relationship in the resultant topologies. Two *Lemur catta *sequences were used as the outgroup.

## Results

### Microsatellite variation

Overall, the multilocus panel employed was found to be very informative. The probability that two individuals cannot be differentiated is 3.08E-9 for *E. mongoz *and 3.64E-14 for *E. fulvus*. With one exception, all individuals have unique multilocus genotypes. One adult mongoose lemur male from Anjamena sampled in 1995 has exactly the same genotype as a male from 1997. Because none of the wild animals sampled were marked permanently, it is likely that the same male was captured twice. Therefore, one of the two samples was removed from the data set.

Variation among mongoose and brown lemurs for the 12 microsatellite loci is summarized in Table 3. There is no evidence for linkage disequilibrium between loci in the mongoose lemur population at Anjamena. When assessing the parent-offspring relationships in the captive mongoose lemur population with known pedigrees, all alleles are found to segregate with Mendelian expectations. Allelic diversity ranges from 3 to 9 alleles per locus (mean = 5.9 ± 1.8 SE) in mongoose lemurs and from 3 to 17 (9.0 ± 3.9) in brown lemurs. Mongoose lemurs at Anjamena have observed heterozygosities of 0.16 to 0.89 (0.50 ± 0.20) per locus, which are slightly higher than expected heterozygosities (Table 3). In the mongoose lemur population at Anjamena, no locus differs significantly from Hardy-Weinberg equilibrium. The other samples were not tested for the Hardy-Weinberg equilibrium because they are either from captivity (no random mating) or from different subspecies (different populations). All loci have low inbreeding coefficients (-0.17<F_is _< 0.40, Table 3). With one exception, estimates of null allele frequencies range from -0.11 to 0.05 for each locus, indicating absence of null alleles. Locus Em5 has an estimated null allele frequency of 0.24. However, since the locus is in Hardy-Weinberg equilibrium and the alleles are inherited in Mendelian fashion when testing the 45 offspring with known parents, the presence of a null allele is unlikely to be the reason for this result. We therefore retained this locus for further data analyses.

**Table 3:** Characteristics* of the 12 microsatellite loci used in *E. mongoz *and *E. fulvus*

**Locus**	***E. mongoz *at Anjamena**					***E. mongoz***			***E. fulvus***		
			
	**N_I_**	**N_A_**	**H_O_**	**H_E_**	**F_is_**	**SR**	**N_I_**	**N_A_**	**SR**	**N_I_**	**N_A_**
Em1	37	5	0.892	0.767	-0.165	161–175	107	8	161–199	53	17
Em2	37	3	0.162	0.153	-0.061	156–164	107	4	150–176	54	11
Em4	37	4	0.568	0.504	-0.129	146–158	107	5	142–160	55	8
Em5	37	2	0.270	0.444	0.395	172–176	107	3	170–180	55	6
Em7	37	6	0.649	0.729	0.112	129–147	107	7	129–151	55	9
Em8	37	6	0.703	0.719	0.023	159–177	107	9	143–175	55	12
Em9	37	4	0.432	0.437	0.011	171–183	106	7	169–197	53	13
Em11	37	4	0.541	0.534	-0.013	250–259	107	6	247–257	55	6
Em15	37	4	0.595	0.652	0.089	206–224	107	6	204–212	55	3
Lc1	37	5	0.757	0.672	-0.129	86–98	107	5	90–100	55	6
Lc8	35	5	0.429	0.393	-0.091	221–231	104	7	217–237	50	11
Efr09	37	4	0.622	0.542	-0.150	99–105	107	4	95–107	51	6

Mean	36.8	4.3	0.552	0.545	-0.009		106.7	5.9		53.8	9.0

* N_I _= number of individuals genotyped; N_A _= number of alleles found; H_O _= observed heterozygosity; H_E _= expected heterozygosity; F_is _= inbreeding coefficient; SR = size range of alleles in base positions

### Species differentiation and hybrids

Analysis of molecular variance applied to the two *Eulemur *species showed 23.21% between-species variability (AMOVA, P < 0.0001), indicating that 76.79% of the genetic variation was found within each species. Genetic differentiation between the two species is relatively high (F_st _= 0.232, R_st _= 0.636) and significant at each locus (exact G test, P = 0).

**Figure 2:**
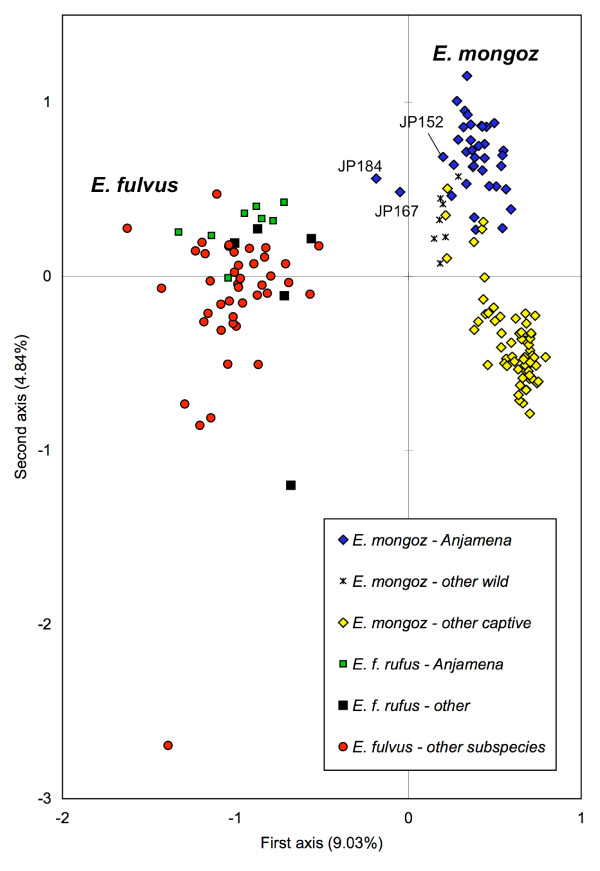
**Principal component analysis of individual *E. mongoz *and *E. fulvus *genotypes**. First and second axes represent the first two factorial components. The two hybrids (JP167 and JP184) are specially marked.

Principal component analysis (PCA) clearly separates brown lemurs from mongoose lemurs (left and right on Figure 2). Within *E. mongoz*, the wild and captive populations are grouped apart (top and bottom in Figure 2) with little overlap. There are no obvious subgroups among brown lemurs. Two mongoose lemurs (JP167 and JP184) are intermediate between *E. mongoz *and *E. fulvus *(Figure 2).

**Figure 3:**
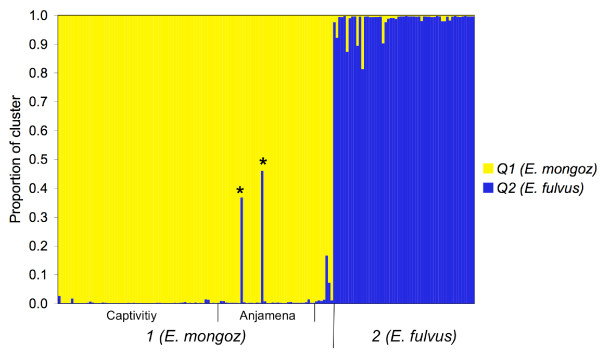
**Admixture analysis of 162 mongoose and brown lemurs**. Each individual is represented by a single vertical line broken into K = 2 segments, with lengths proportional to the estimated membership in each cluster (Q1 for *E. mongoz *and Q2 for *E. fulvus*). The two hybrids (JP167 and JP184) are marked with *.

We used STRUCTURE on the microsatellite data to identify distinct genetic populations, assign individuals to populations, and identify admixed individuals. When assuming two populations (K = 2), all 162 samples are correctly assigned to their species (Figure 3). Most mongoose lemurs clearly fall into cluster 1 with the proportion of membership Q1 ranging from 0.833 to 0.998 (0.994 ± 0.018). Only two mongoose lemurs from Anjamena (JP167 and JP184) split between cluster 1 (Q1 = 0.632 and 0.539) and cluster 2 (Q2 = 0.368 and 0.461). All 55 brown lemurs are grouped into cluster 2, with Q2 ranging from 0.814 to 0.998 (0.983 ± 0.035).

When K is increased from 2 up to 16, the samples are divided into more clusters, with K = 12 showing the highest probability. The different clusters mainly represent subspecies in *E. fulvus *or populations/groups in mongoose lemurs (data not shown). However, while individuals might be admixed between populations or subspecies of the same species, there is no admixture of the two species (apart from JP167 and JP184). These tests confirm the clear separation of the two species and the intermediate positioning of JP167 and JP184.

We also ran analyses using only the 42 mongoose and brown lemurs from Anjamena. When assuming K = 2 populations, most lemurs are clearly assigned to the correct species (Q1 = 0.996 ± 0.006 in most *E. mongoz *and Q2 = 0.995 ± 0.004 in all *E. fulvus*). There are 3 mongoose lemurs that are split between the two clusters. As in the previous analyses, the two individuals JP167 and JP184 have the highest probabilities to be admixed (Q1/Q2 = 0.527/0.473 and 0.714//0.286). In addition, a third mongoose lemur (JP152) has a slightly increased proportion of membership for cluster 2 (Q2 = 0.192). When increasing K, no further subdivision at Anjamena is possible.

### Introgression of microsatellite alleles in mongoose lemurs

A total of 71 alleles are found at the 12 loci in the 107 mongoose lemurs studied. The 37 *E. mongoz *at Anjamena have 53 different alleles, of which 10 alleles are not found in any other mongoose lemur outside Anjamena. Assuming that hybridization is restricted to Anjamena, these 10 private alleles for Anjamena are potential candidates for introgression into the mongoose lemur gene pool by hybridization with brown lemurs. However, one allele is not observed in any brown lemur, which excludes it from being of *E. fulvus *origin. Details of the remaining 9 alleles are given in Table 4.

**Table 4:** Details on 15 microsatellite alleles, which might originate from the other species

			***E. mongoz***			***E. fulvus***		
				
**Origin**	**Locus**	**Allele**	**N^a^**	**F^b^**	**Other^c^**	**N^a^**	**F^b^**	**Other^c^**
*Fulvus*	Em11	251	1	0.01		2	0.13	+
	Em11	253	1	0.01		13	0.81	+
	Em2	164	1	0.01		1	0.06	+
	Em7	129	1	0.01		1	0.06	+
	Lc8	223	1	0.01		0	-	+
	Em15	206	2	0.03		14	0.88	+
	Lc1	98	3	0.04		5	0.31	+

*Fulvus*^d^	Em4	146	1	0.01		7	0.44	+
	Lc8	221	5	0.07		3	0.21	+

*Mongoz*	Em1	163	12	0.16	+	9	0.64	
	Em1	167	23	0.30	+	2	0.14	+
	Em8	159	19	0.23	+	2	0.13	+
	Em9	179	2	0.03	+	3	0.19	+
	Lc1	94	19	0.24	+	2	0.13	+

^a ^Number of alleles found at Anjamena

^b ^Allele frequency at Anjamena

^c ^+ indicates that the allele is also found in individuals outside Anjamena

^d ^Those two alleles are hybrid candidates for both taxa.

Five of the possibly introgressed alleles are very common (>20%) in *E. f. rufus *at Anjamena, which supports their origin in brown lemurs. Three alleles are found only once or twice among the 8 Anjamena brown lemurs (6–13%), however, since they are very rare in mongoose lemurs (each allele only found once), they might still be of brown lemur origin. One mongoose lemur at Anjamena has an allele (Lc8–223), not found in any other mongoose lemur and also not in the brown lemurs at Anjamena. Only one *E. f. fulvus *has that same allele.

The two hybrid mongoose lemurs (JP167 and JP184) identified from PCA/STRUCTURE analyses have 4 putative introgressed alleles each. These two animals have 7 putative introgressed alleles found only once or twice in the mongoose lemurs at Anjamena (Table 4). Another 6 mongoose lemurs at Anjamena have one or both of the remaining 2 possibly introgressed alleles. One allele (Lc1–98) occurs in 3 mongoose lemurs and with a frequency of 31% is well represented among the brown lemurs in Anjamena. The other allele (Lc8–221) is found 5 times in mongoose lemurs and has an allele frequency of 21% in *E. f. rufus*.

### Parentage of hybrids

At the time of our field study, JP167 was still a juvenile female without offspring. The other hybrid JP184 was an adult female. We were able to collect samples from the adult male and the juvenile of her social group. In parentage testing using microsatellites, neither the adult male nor the hybrid female JP184 are excluded as parents of the juvenile JP152. With a high probability (99.0% if accepting the adult male as the father, 91.0% if only considering the female), this makes JP184 the mother of the juvenile.

### Introgressed microsatellite alleles in brown lemurs

**Figure 4:**
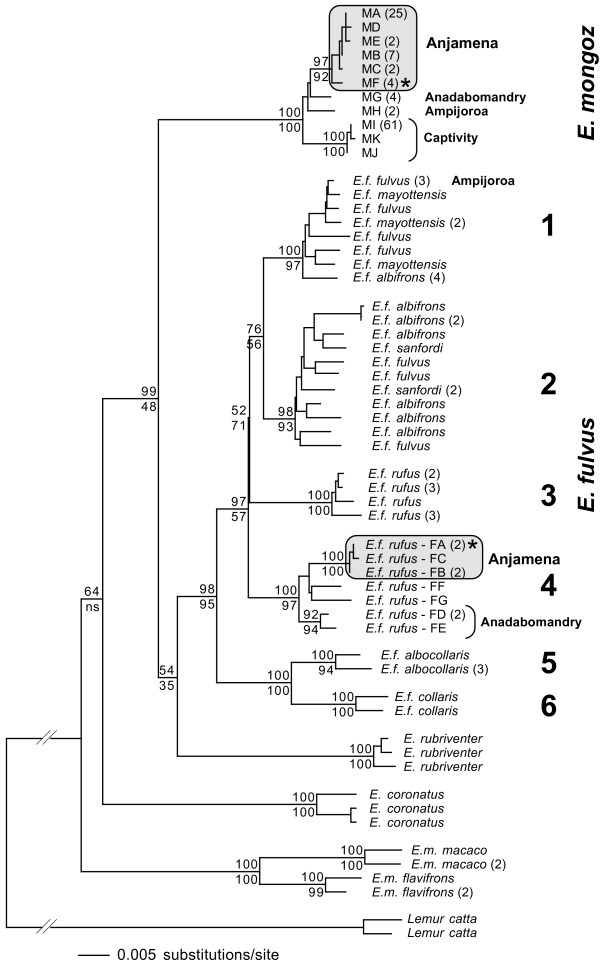
**Neighbor-joining phylogram of 176 lemurs sequenced for a D-loop and ND4 fragment of the mtDNA genome**. Clades containing animals with identical haplotypes were lumped together into one branch, providing the number of united individuals behind the taxon name. The two haplotypes of *E. mongoz *and *E. fulvus*, which were also found in the other species, are marked with an asterisk (*). Bootstrap values obtained with neighbor-joining (above nodes) and maximum parsimony (below nodes) analyses are provided at relevant branches. "ns" means that the maximum parsimony tree topology did not show that node.

The 13 brown lemurs in the *E. f. rufus *subspecies clade, which includes the animals from Anjamena (see Figure 4, the other 9 *E. f. rufus *samples mentioned in Table 1 form another subspecies clade), have 60 different alleles. Seven alleles are unique to the 8 brown lemurs at Anjamena and also present in the mongoose lemur gene pool at Anjamena. Two of these alleles are only found in mongoose and brown lemurs at Anjamena and hence could originate from either species (Em4–146 and Lc8–221, Table 4). Based on their low frequency in mongoose lemurs and high frequency in brown lemurs they are more likely to be of brown lemur origin. This leaves 5 alleles, which might have been introduced into the brown lemur gene pool by hybridization (Table 4). With one exception, each brown lemur has 1 to 5 of these possibly introgressed alleles in its haplotype (2.57 ± 1.51). Due to the very low sample size for *E. f. rufus *(N = 13), this identification of putatively introgressed alleles is only preliminary.

### mtDNA results

We sequenced two fragments of the mtDNA genome for 107 mongoose lemurs and 55 brown lemurs, as well as 12 individuals from the other three *Eulemur *species and 2 *Lemur catta*. The aligned nucleotide sequences span a total of 1463 base positions (bp). The analysed data set consists of 31 bp of tRNA^Thr^, 66 bp of tRNA^Pro^, 804 bp of D-loop and 559 bp of ND4. Details on the number of haplotypes found with D-loop data, ND4 data and the combined data set in animals at Anjamena or across the species range are given in Table 5. A total of 36 variable positions are present among mongoose lemurs, while brown lemurs exhibit 178 variable sites (Table 5). Nucleotide divergence between the two species ranges from 0.054 to 0.077 (0.063 ± 0.004 Tamura-Nei distance). Some of the ND4 sequences have been published previously [27,43]. New sequences were deposited on GenBank [D-loop: EU333172–EU333247, ND4: EU333248–EU333274, see also Additional file 1].

**Table 5:** mtDNA haplotypes found in mongoose lemurs and brown lemurs

	***E. mongoz***		***E. fulvus***		
		
	**Anjamena**	**All**	**Anjamena**	***E. f. rufus*^a^**	**All**
# individuals	37	107	8	13	55
# haplotypes D-loop	2+1*	5+1*	1+1*	5+1*	31+1*
# haplotypes ND4	4+1*	9+1*	3+1*	6+1*	30+1*
# haplotypes D-loop & ND4	5+1*	10+1*	3+1*	7+1*	34+1*
# variable base positions D-loop^b^	1	25	0	29	114
# variable base positions ND4^b^	3	11	2	8	64

^a ^only brown lemurs from the same subspecies clade (clade #4 in Figure 4)

^b ^excluding the haplotype from the other species

* one haplotype was found from the other *Eulemur *species

There are 5 mtDNA haplotypes (MA-ME) in the mongoose lemur population at Anjamena. In addition, one mongoose lemur exhibits the mtDNA haplotype of a brown lemur. The brown lemurs at Anjamena have 3 haplotypes (FA, FB, FC). One of these (FA), is the haplotype also observed in the mongoose lemur. There are 4 *E. fulvus *at Anjamena with the *E. mongoz *haplotype MF, which is not found in any mongoose lemur at Anjamena. While the haplotypes MA-ME differ from each other by 1 or 2 bp, haplotype MF is 4–5 bp different from the other haplotypes found among mongoose lemurs in Anjamena. The 3 *E. fulvus *haplotypes at Anjamena differ from each other by 1 or 2 bp.

Among all 107 mongoose lemurs, 10 haplotypes (MA-MK) are found. The most common (57%) haplotype, MI, is present in 61 *E. mongoz *kept in various zoos. Two animals in captivity each have a unique haplotype (MJ and MK). The 3 haplotypes found in captivity differ from each other by only 1 or 2 bp. The two animals at Ampijoroa have haplotype MH and the 4 mongoose lemurs from Anadabomandry have haplotype MG.

In the phylogenetic analyses, four distinct clades of mongoose lemurs can be recognized (Figure 4). One clade contains the animals from Anjamena, including the haplotype found in the 4 brown lemurs at Anjamena. The 3 haplotypes from the captive mongoose lemurs form a second clade while the haplotypes from Ampijoroa and Anadabomandry each form a separate clade. Haplotypes between those 4 clades differ from each other at 12 to 21 bp (17.6 ± 3.2).

Among brown lemurs, a total of 34 haplotypes are found. Phylogenetic analyses group them into 6 clades (Figure 4). Each of these subspecies clades gets 93 – 100% bootstrap support in neighbor-joining and maximum parsimony analyses (Figure 4). The clade containing the brown lemurs from Anjamena includes 5 additional animals, which add 4 more haplotypes to this clade: Two haplotypes FD and FE are present at Anadabomandry, which differ from each other by 5 bp. One zoo animal of unknown origin also exhibits haplotype FD. The remaining 2 haplotypes are found in a zoo animal of unknown origin (FF) and in a red-fronted brown lemur from Maintirano, which lies further south from Anjamena on the western coast of Madagascar (FG).

## Discussion

### F1 hybrids in the mongoose lemur population

Principal component analysis positions two animals between *E. mongoz *and *E. fulvus *(Figure 2) and admixture analyses reveal an admixed genotype for the same two individuals (Figure 3). These two animals are most likely F1 hybrids. Whereas one allele per locus and, hence, 12 introgressed alleles would be expected in an F1 hybrid, we observed only 4. The 8 brown lemurs from Anjamena have a total of 37 different alleles in their genotypes, of which 16 alleles are shared with the mongoose lemurs at Anjamena. Therefore, only 21 alleles are unique to *E. fulvus *and are identifiable as introgressed in a hybrid. Taking the allele frequencies into account, a hybrid would inherit from its brown lemur parent on average 5.03 alleles which are exclusive to brown lemurs and 6.97 alleles which are shared between *E. mongoz *and *E. fulvus*, which is consistent with the pattern found.

JP167 is a juvenile female, which is part of a group consisting of 3 animals. Fur coloration of JP167 is clearly intermediate between brown lemur and mongoose lemur females. This led us to suspect in the field, that she might be a hybrid. The two other group members are an adult male and an adult female. We were only able to capture the adult female, that exhibited normal mongoose lemur fur coloration. She has the mtDNA haplotype of a regular mongoose lemur and has no putative introgressed alleles. Apart from the mtDNA, this adult female is also excluded at 2 microsatellite loci from being the mother of JP167.

The second mongoose lemur of hybrid origin is an adult female (JP184). She is a member of a small social group, including herself, an adult male, subadult female and juvenile male. The adult male has the normal fur coloration of a mongoose lemur male. The juvenile (JP152), like JP184 exhibits a mixture of fur colours from *E. mongoz *and *E. fulvus*. Parentage testing indicates that the adult hybrid female JP184 is most likely (>90%) the mother of the juvenile. The genotype of the juvenile contains none of the 4 putative introgressed alleles from the mother. However, admixture analyses of the lemurs at Anjamena reveal a proportion of brown lemur ancestry (Q2 = 0.192) in its genotype that is consistent with an F2.

### Descendants of hybrids in the mongoose lemur population

The 6 mongoose lemurs with one or two putative introgressed alleles might be descendants of a hybrid crossing. Neither PCA nor admixture analyses were able to differentiate them from other mongoose lemurs. Consistent with this, their coat coloration was normal for mongoose lemurs and so they could not be F1s, and are unlikely to be F2s, since the confirmed offspring of an F1 hybrid and a mongoose lemur (JP152) has intermediate coat colour. If hybridization had occurred, a single event could have accounted for the allele distribution. Further data would be required to assess whether these alleles truly represent a signature of past introgression events, and to estimate the rate of introgression.

### Conclusions on hybrid crossings in the mongoose lemur population

We found genetic evidence for a minimum of 2 hybridization events in mongoose lemurs of Anjamena. Two hybrid crossings were very recent (F1). In addition, one of the F1 hybrids had an offspring where paternity could be genetically confirmed. Therefore, hybridization between mongoose and brown lemurs occurs in the wild and yields fertile offspring, which can successfully backcross with mongoose lemurs. A third potential hybrid crossing might have happened a few generations ago, which would again show the fertility of the hybrids.

One of the F1 hybrids has the mtDNA haplotype of a brown lemur and the other of a mongoose lemur. Hybrid crossings can therefore occur between *E. mongoz *females and *E. fulvus *males as well as between *E. mongoz *males and *E. fulvus *females.

### Introgression into the brown lemur population

Due to the small sample size (N = 8) and also not knowing the familial relationships among the members of each social group (containing several males and females), no detailed information on hybrid crossings can be obtained for the brown lemurs at Anjamena. However, half of the brown lemurs sampled have an *E. mongoz *mtDNA haplotype. As all 4 animals have the same haplotype MF, the presence of that haplotype could be the consequence of a single hybrid crossing. Interestingly, haplotype MF is not found in any of the 35 mongoose lemurs sampled at Anjamena and is the most divergent of the 5 *E. mongoz *haplotypes found there (Figure 4). These two factors make it likely that the hybrid crossing from which haplotype MF is derived occurred in the past, with the mongoose lemurs at Anjamena subsequently losing haplotype MF due to genetic drift. Alternatively, it could have occurred between a brown lemur male and a mongoose lemur female located some distance from Anjamena.

Seven of the eight brown lemur genotypes from Anjamena contain putative introgressed microsatellite alleles. The only brown lemur without any introgressed alleles has the mtDNA genotype of *E. mongoz*. The samples were collected from animals living in 7 different social groups at Anjamena. Principal component analysis failed to group the 8 animals closely together (Figure 2). Therefore, close familial relationships are no explanation for the high presence of introgressed alleles among these brown lemurs. Fur coloration of all 8 animals appeared to be normal for red-fronted brown lemurs, excluding them from being F1 or F2 hybrids. However, a larger sample from brown lemurs at Anjamena and across the subspecies range is needed to confirm this result. Some of the current putative introgressed alleles might be found in other *E. f. rufus *outside Anjamena, making an origin in *E. mongoz *unlikely, while a more detailed study on the brown lemurs at Anjamena might reveal new introgressed alleles.

### Evolutionary consequences

Some time after the five species of *Eulemur *radiated across Madagascar, a brown lemur radiation started, most likely in the South, where *E. f. albocollaris *and *E. f. collaris *occur. This is the only area where brown lemurs do not overlap with another *Eulemur *species. Brown lemurs then radiated back across the island to the North [43], becoming sympatric with the other 4 *Eulemur *species. However, no hybrids are reported between the brown lemurs and sympatric *E. rubriventer *in the East, *E. coronatus *in the North, or *E. macaco *in the Northwest. *E mongoz *is sympatric with *E. f. fulvus *and *E. f. rufus *in the West, but there also seems to be no hybridization between *E. mongoz *and *E. f. fulvus*. Only *E. f. rufus *and *E. mongoz *at Anjamena are known to hybridize.

A possible explanation for this hybridization is that one species only recently expanded its range and there were no reproductive barriers in place between the two newly overlapping taxa. During the radiation of brown lemurs the Betsiboka river formed a barrier dividing *E. fulvus *populations, resulting in it being the northern limit of *E. f. rufus*, with *E. f. fulvus *subspecies being formed across the river (see also Figure 1). In contrast, *E. mongoz *is one of the few lemur taxa for which the Betsiboka river is not a taxonomic boundary [44]. The current mongoose lemur range extends a little southwest of Anjamena, across the Mahavavy river. Our mtDNA sequence data shows (Figure 4) that the mongoose lemurs at Anjamena are substantially different from those at Ampijoroa (14–15 bp out of 1463 bp), but do not differ to the extent seen in the two brown lemur subspecies (44–46 bp). This implies that the Betsiboka river serves as a geographic boundary in both taxa, but was crossed by *E. mongoz *later. The other option would be that *E. f. rufus*, coming from the southwest, only recently crossed the Mahavavy river. However, genetic distances between animals on the west (Anadabomandry) and east (Anjamena) side of this river are higher for *E. f. rufus *(21–24 bp) than for *E. mongoz *(12–15 bp). This again indicates that *E. mongoz *expanded its range more recently than *E. f. rufus*.

A cladistic biogeographic analysis on the extant lemur distribution in western Madagascar suggests that *E. mongoz *either had a wide distribution in the past and then disappeared from large parts of its former range, or dispersed across otherwise efficient geographical barriers [45]. In 1939, Lamberton found a subfossil femur of *E. mongoz *at Ampasambazimba [46], which lies in the central western highlands of Madagascar, where the Betsiboka and Mahavavy rivers originate. More subfossil mongoose lemur remains were found near the coast at Anjohibe [46], to the east of the Betsiboka river (Figure 1). There are two possible scenarios, which would explain the unusual current mongoose lemur distribution [45], the fossil evidence thus far available [46] and the low genetic differentiation across the Betsiboka and Mahavavy rivers in *E. mongoz *(this study). One is, that mongoose lemurs had a large distribution in the past with regular gene flow across the range. Only once they lost their range in central Madagascar relatively recently, was gene flow no longer possible across the range because the Betsiboka and Mahavay rivers became geographical barriers. The other option is, that in the past mongoose lemurs occurred only to the east of the Betsiboka river all the way up to central highlands and only later expanded their range towards the coast on the west side of the Betsiboka and Mahavavy rivers, while disappearing from central Madagascar. In the first scenario they were sympatric with *E. f. fulvus *and *E. f. rufus *for a long time, while in the second scenario they only became sympatric with *E. f. rufus *relatively recently.

The influx of a new species into a novel ecosystem can result in hybridization between the new species (in this case *E. mongoz*) and the related native species (*E. fulvus*). Where few reproductive barriers to gene flow exist, this frequently leads to the rapid introgression of genetic and phenotypic characters from one species into another [47]. In captivity, crosses between various *E. fulvus *subspecies resulted in fertile offspring [48]. Among captive *Eulemur *species, crossbreeding between *E. macaco *and *E. fulvus *gave rise to fertile hybrids [48]. In the wild, hybridization has been reported between *E. macaco macaco *and *E. m. flavifrons *[16], *E. f. fulvus *and *E. f. rufus *[17], and *E. f. rufus and E. f. albocollaris *[18] subspecies. All these hybridization events among *Eulemur *species and subspecies indicate that reproductive barriers among *Eulemur *taxa are not very well developed.

Hybridization between newly overlapping species provides a unique opportunity to observe the initial stages of hybridization and its evolutionary consequences. Hybridization which results in fertile offspring, is expected to introduce sets of alleles that gradually disperse through the gene pool of the parent organisms through successive backcrosses. Such extensive introgression might have occurred among brown lemurs at Anjamena with 7 (88%) brown lemurs possibly having introgressed alleles and 4 (50%) brown lemurs having the mtDNA haplotype of *E. mongoz*. However, larger sample sizes are needed to confirm the extent of introgression. In mongoose lemurs, only 8 (22%) of the 37 animals studied show signs of introgression.

There are several possible evolutionary consequences of hybridization (see also introduction). In extreme cases, parental taxa may be lost in the process and/or new taxa formed [21,22]. A third possibility is that a stable hybrid zone will form, with limited introgression across the zone. Having already radiated across Madagascar twice, the genus *Eulemur *has proved to be very successful in adapting to a variety of ecological niches. Introgressive hybridization among taxa is known to quickly increase levels of variation, allowing more rapid responses to environmental changes [21,22]. Considering all the reported hybridization events known so far between *Eulemur *taxa in the wild [[16-18], this study], hybridization might also have played an important role in the success of the radiation of *Eulemur *across Madagascar.

## Conclusion

The genetic data presented here confirms the occurrence of hybridization between *E. mongoz *and *E. fulvus *in western Madagascar. Most other hybrid zones reported so far in lemurs are also between *Eulemur *taxa [16-18]. Only one hybridization event has been found among other lemur taxa, that of *Varecia variegata *subspecies [19]. It is known that some groups of organisms seem to hybridize more readily than others [1,47] and *Eulemur *may be more capable of hybridizing than other lemur taxa. However, hybrids are not easy to detect. They are much easier to identify and are generally recorded more often if the hybridizing taxa are brightly and distinctively coloured (e.g., birds, butterflies [1]). That is very much the case for *Eulemur *species and subspecies, as well as for *Varecia variegata *subspecies. The detection of hybridization in lemurs may follow a similar time sequence as the discovery of parent taxa, with nocturnal species lagging behind. For nocturnal lemurs, the pelage coloration of which is neither bright nor varies much between taxa, only detailed genetic studies are likely to detect hybridization events. Our study records an instance of on-going hybridization in the wild. Introgressive hybridization may hasten speciation and allow rapid ecological adaptation of taxa, hence be one of the driving forces for the adaptive radiation of lemurs in Madagascar.

## Authors' contributions

JP participated in the design of the study and the field expedition, carried out the lab work, conducted data analyses and wrote the first draft of the manuscript. AZ organized and lead the field expedition to Anjamena to collect the samples. DC participated in the design of the study, helped with the logistics of the field expedition and revised a draft version of the manuscript. CN advised and participated in the data analyses. NM critically revised the draft version of the manuscript. All authors approved the final version of the manuscript.

## Supplementary Material

Additional File 1 **Locality, sample type and GenBank numbers for all *E. mongoz *haplotypes and *E. fulvus *samples.** none. Click here for file
