# Supplementary material for: Genetic analysis of hybridization and introgression between wild mongoose and brown lemurs
Source: BMC Evol Biol. 2009 Feb 5;9:32. doi: 10.1186/1471-2148-9-32 (PMC2657121; doi:10.1186/1471-2148-9-32)
Supplement: Additional File 1 — Locality, sample type and GenBank numbers for all E. mongoz haplotypes and E. fulvus samples. none. [file 1471-2148-9-32-S1.doc]

**Additional file 1.** Locality, sample type and GenBank numbers for all *E. mongoz* haplotypes and *E. fulvus* samples.

| **Taxon** | **Locality** | **ID** | **Sample** | **GenBank#** | **GenBank#** |
| --- | --- | --- | --- | --- | --- |
|  |  |  |  | **D-loop** | **ND4** |
| *E. mongoz* | Anjamena (N=25) | MA | Blood | EU333172 | EU333248 |
| *E. mongoz* | Anjamena (N=7) | MB | Blood | EU333173 | EU333249 |
| *E. mongoz* | Anjamena (N=2) | MC | Blood | EU333174 | EU333250 |
| *E. mongoz* | Anjamena (N=1) | MD | Blood | EU333175 | EU333251 |
| *E. mongoz* | Anjamena (N=2) | ME | Blood | EU333176 | EU333252 |
| *E. mongoz* | Anadabomandry (N=4) | MG | Blood | EU333178 | EU333254 |
| *E. mongoz* | Ampijoroa (N=2) | MH | Hair | EU333179 | AF224519 |
| *E. mongoz* | Captivity (N=61) | MI | Blood, hair, tissue | EU333180 | EU333255 |
| *E. mongoz* | Köln Zoo (N=1) | MJ | Tissue | EU333181 | AF224521 |
| *E. mongoz* | Cricket St. Thomas Zoo | MK | Blood | EU333182 | EU333256 |
| *E. f. albifrons* | Berlin Zoo | JP2 | Hair | EU333183 | AF224564 |
| *E. f. albifrons* | Zoo Mulhouse | JP25 | Hair | EU333184 | AF224565 |
| *E. f. albifrons* | Zoo Mulhouse | JP26 | Hair | EU333185 | EU333257 |
| *E. f. albifrons* | Zoo Mulhouse | JP35 | Hair | EU333192 | EU333258 |
| *E. f. albifrons* | Tsimbazaza Zoo | JP45 | Hair | EU333186 | EU333259 |
| *E. f. albifrons* | Tsimbazaza Zoo | JP134 | Blood | EU333193 | AF224567 |
| *E. f. albifrons* | Tsimbazaza Zoo | JP135 | Blood | EU333187 | AF224566 |
| *E. f. albifrons* | Andranobe River / Forest | JP323 | Tissue | EU333188 | AF224568 |
| *E. f. albifrons* | Ruhr Zoo Gelsenkirchen | JP408 | Hair | EU333189 | EU333260 |
| *E. f. albifrons* | Ruhr Zoo Gelsenkirchen | JP409 | Hair | EU333190 | EU333261 |
| *E. f. albifrons* | Ruhr Zoo Gelsenkirchen | JP410 | Hair | EU333191 | EU333262 |
| *E. f. albocollaris* | Tsimbazaza Zoo | JP145 | Blood | EU333228 | AF224562 |
| *E. f. albocollaris* | Université Louis Pasteur | JP222 | Blood | EU333229 | AF224558 |
| *E. f. albocollaris* | Université Louis Pasteur | JP223 | Blood | EU333230 | EU333273 |
| *E. f. albocollaris* | Université Louis Pasteur | JP224 | Blood | EU333231 | EU333274 |

| **Taxon** | **Locality** | **ID** | **Sample** | **GenBank#** | **GenBank#** |
| --- | --- | --- | --- | --- | --- |
|  |  |  |  | **D-loop** | **ND4** |
| *E. f. collaris* | Fort Dauphin (pet) | JP304 | Hair | EU333232 | AF224559 |
| *E. f. collaris* | Banham Zoo | JP307 | Tissue | EU333233 | AF224560 |
| *E. f. fulvus* | Tsimbazaza Zoo | JP41 | Hair | EU333195 | AF224534 |
| *E. f. fulvus* | Ampijoroa | JP215 | Blood | EU333196 | AF224535 |
| *E. f. fulvus* | Ampijoroa | JP216 | Blood | EU333197 | EU333263 |
| *E. f. fulvus* | Ampijoroa | JP218 | Hair | EU333198 | AF224536 |
| *E. f. fulvus* | Ampijoroa | JP329 | Blood | EU333199 | EU333264 |
| *E. f. fulvus* | Antsohihy (pet) | JP330 | Blood | EU333200 | AF224537 |
| *E. f. fulvus* | Antsohihy (pet) | JP331 | Blood | EU333194 | AF224538 |
| *E. f. fulvus* | Tsimbazaza Zoo | JP336 | Blood | EU333201 | AF224539 |
| *E. f. fulvus* | Tsimbazaza Zoo | JP337 | Blood | EU333202 | AF224540 |
| *E. f. mayottensis* | Leipzig Zoo | JP72 | Hair | EU333203 | AF224541 |
| *E. f. mayottensis* | Université Louis Pasteur | JP225 | Blood | EU333204 | AF224542 |
| *E. f. mayottensis* | Université Louis Pasteur | JP226 | Blood | EU333205 | AF224543 |
| *E. f. mayottensis* | Leipzig Zoo | JP440 | Hair | EU333206 | EU333265 |
| *E. f. rufus* | Tsimbazaza Zoo | JP43 | Hair | EU333207 | EU333266 |
| *E. f. rufus* | Tsimbazaza Zoo | JP124 | Blood | EU333208 | EU333267 |
| *E. f. rufus* | Anjamena | JP161 | Blood | EU333209 | AF224545 |
| *E. f. rufus* | Anjamena | JP162 | Blood | EU333177 | EU333253 |
| *E. f. rufus* | Anjamena | JP171 | Blood | EU333210 | AF224547 |
| *E. f. rufus* | Anadabomandry | JP176 | Blood | EU333222 | AF224548 |
| *E. f. rufus* | Anjamena | JP179 | Blood | EU333177 | EU333253 |
| *E. f. rufus* | Anadabomandry | JP181 | Blood | EU333223 | AF224549 |
| *E. f. rufus* | Anjamena | JP189 | Hair | EU333177 | EU333253 |
| *E. f. rufus* | Anjamena | JP190 | Hair | EU333177 | EU333253 |
| *E. f. rufus* | Anjamena | JP192 | Blood | EU333211 | EU333268 |
| *E. f. rufus* | Anjamena | JP206 | Blood | EU333212 | AF224550 |

| **Taxon** | **Locality** | **ID** | **Sample** | **GenBank#** | **GenBank#** |
| --- | --- | --- | --- | --- | --- |
|  |  |  |  | **D-loop** | **ND4** |
| *E. f. rufus* | Unknown | JP328 | Blood | EU333224 | EU333269 |
| *E. f. rufus* | Morondava | JP332 | Blood | EU333213 | AF224551 |
| *E. f. rufus* | Maintirano | JP333 | Blood | EU333214 | AF224552 |
| *E. f. rufus* | Tsimbazaza Zoo | JP334 | Blood | EU333215 | EU333270 |
| *E. f. rufus* | Tsimbazaza Zoo | JP335 | Blood | EU333216 | EU333271 |
| *E. f. rufus* | Tsimbazaza Zoo | JP338 | Blood | EU333217 | AF224553 |
| *E. f. rufus* | Tsimbazaza Zoo | JP339 | Blood | EU333218 | AF224554 |
| *E. f. rufus* | Tsimbazaza Zoo | JP340 | Blood | EU333219 | AF224555 |
| *E. f. rufus* | Tsimbazaza Zoo | JP341 | Blood | EU333220 | AF224556 |
| *E. f. rufus* | Tsimbazaza Zoo | JP342 | Blood | EU333221 | AF224557 |
| *E. f. sanfordi* | Tsimbazaza Zoo | JP40 | Hair | EU333227 | EU333272 |
| *E. f. sanfordi* | Tsimbazaza Zoo | JP125 | Blood | EU333225 | AF224563 |
| *E. f. sanfordi* | Tsimbazaza Zoo | JP126 | Blood | EU333226 | AF224561 |
| *E. coronatus* | Mulhouse Zoo | JP33 | Hair | EU333234 | AF224522 |
| *E. coronatus* | Mulhouse Zoo | JP34 | Hair | EU333235 | AF224523 |
| *E. coronatus* | Tsimbazaza Zoo | JP121 | Blood | EU333236 | AF224524 |
| *E. macaco macaco* | Université Louis Pasteur | JP80 | Blood | EU333240 | AF224528 |
| *E. macaco macaco* | Université Louis Pasteur | JP82 | Blood | EU333241 | AF224529 |
| *E. macaco macaco* | Université Louis Pasteur | JP83 | Blood | EU333242 | AF224530 |
| *E. m. flavifrons* | Université Louis Pasteur | JP74 | Blood | EU333243 | AF224531 |
| *E. m. flavifrons* | Université Louis Pasteur | JP75 | Blood | EU333244 | AF224532 |
| *E. m. flavifrons* | Université Louis Pasteur | JP77 | Blood | EU333245 | AF224533 |
| *E. rubriventer* | Tsimbazaza Zoo | JP129 | Blood | EU333237 | AF224525 |
| *E. rubriventer* | Tsimbazaza Zoo | JP130 | Blood | EU333238 | AF224526 |
| *E. rubriventer* | Université Louis Pasteur | JP229 | Blood | EU333239 | AF224527 |
| *Lemur catta* | Zürich Zoo | JP3 | Tissue | EU333246 | AF053684 |
| *Lemur catta* | Zürich Zoo | JP52 | Hair | EU333247 | AF224570 |
